# Modeling the effects of physical activity, education, health, and subjective wealth on happiness based on Indonesian national survey data

**DOI:** 10.1186/s12889-022-13371-x

**Published:** 2022-05-13

**Authors:** Bhina Patria

**Affiliations:** Faculty of Psychology, Universitas Gadjah Mada, Yogyakarta, Indonesia

**Keywords:** Happiness, Health, Physical activity

## Abstract

**Background:**

Studies on physical activity’s psychological benefits are generally fewer than those on its physiological benefits, and these limited studies have mostly investigated its impact on cognitive functions. Studies exclusively investigating physical activity’s effects on happiness are rare. This study aims to investigate the effect of physical activity on psychological functions, especially on happiness.

**Methods:**

Analysis was based on a large field of nationally representative Indonesian adult data. Data were compiled based on face-to-face interviews with 12,051 adults. Participants provided measures of physical activity, subjective health, and happiness, and responses were recorded with computer-assisted personal interviewing (CAPI) software. Demographic data, including gender, subjective wealth, education, and age, were also included in the analysis. Structural equation modeling (SEM) was conducted to determine the relationship between physical activity, health, subjective wealth, and happiness.

**Results:**

The tested model of the association between physical activity, health, subjective wealth, and happiness indicated a good fit, based on *χ*^2^ (1, *n* = 12,051) = 48.733, *p* = .001, RMSEA = .063, and CFI = .97. Path analysis results showed that health conditions mediated the effects of physical activity on happiness. The result also showed positive effects of education level and subjective wealth on happiness.

**Conclusion:**

This study provides evidence that engagement in physical activity has a positive impact on happiness. Indonesian adults should engage in more active lifestyles since more than one-third of Indonesians did not get enough physical activity.

## Background

Studies on the physiological benefits of physical activity outnumber those of its psychological benefits [1]. Various studies show that regular physical activity has multiple physiological benefits. The intensity of physical activity contributes to lipoprotein profile, carbohydrate metabolism, lower blood pressure, and weight loss [2]. Physical activity also offers protection against cancers of the colon, breast endometrium, pancreas, prostate, lung, and ovary [3–6]. Other studies reported that physical activity helped control type II [7–9] and type I diabetes [10–13]. Blair [14] even concluded that low cardiorespiratory fitness (CRF) was the highest cause of death than other factors—i.e., high blood pressure, smoking, high cholesterol, diabetes, and obesity.

Studies that reported the psychological effects of physical activity were mostly concentrated on cognitive functions. The effects of regular activity were observed across a variety of cognitive processes. However, the highest were found in the executive control process—i.e., planning, scheduling, working memory, interference control, and task coordination [15–17].

In aging adults, regular physical activity maintains cognitive condition and was associated with the decreasing risk of poor cognition and early cognitive decline [18–20]. Weuve and colleagues [19] studied the effects of long-term regular physical activity, mainly walking, on cognitive functions of women aged 70–81 (*N* = 18,766). Results showed that higher levels of regular physical activity were associated with better cognitive performance. Higher cardiorespiratory fitness was also associated with less memory decline across lifespan [19, 21].

Physical activity was also proven to reduce depression [22–25], reduce anxiety [23, 26, 27], and protect against stress [28]. A randomized experiment by Babyak and colleagues [22] showed that exercise was as effective as pharmacotherapy for depression. Furthermore, after 10 months, the exercise group had significantly lower relapse rates than the medication group [22].

The present study investigates the effect of physical activity on psychological functions, especially on subjective wellbeing or happiness. The terms *subjective wellbeing* and *happiness* are used interchangeably in the literature [29]. This study uses the term *happiness* because *wellbeing* usually consists of objective variables such as income and health [29–31]. Happiness in this study refers to the overall evaluation of life or life-satisfaction [32], rather than positive emotional states like contentment, joy, and excitement.

Studies examining the effects of physical activity exclusively on happiness are rare [33–36]. The World Database of Happiness [37], an online register of research on happiness, listed few studies that associated physical activity with happiness. A study on adolescents’ samples concluded that regular exercise was associated with psychological wellbeing and a lower propensity for eating disorder behaviors [38]. Another study on older adults found that physical exercise programs reduced pain intensity [39]. Participants also reported significant improvement in psychological wellbeing—feeling happier, less lonely, more life satisfaction, and less depression [39]. A study found that the intensity of physical activity was positively related to daily positive affect, which is a known determinant of happiness [40].

Why does physical activity affect happiness? Argyle [29] suggested it is partly due to social interaction with others. Physical activity can increase the opportunity for social relationships, which are noted to have a powerful effect on happiness [29]. Nevertheless, physical activity’s effect on happiness was also found in solitary exercise. Literature has long noted the “runner’s high” phenomena, which is suspected to be caused by the release of endorphins after strenuous physical activity [41]. A study measured the activity of the brain before and after strenuous physical activity. The results showed that a release of endogenous opioids occurred in the frontolimbic brain regions after running, and the level of euphoria was significantly increased [42].

The present study also addressed the effects of other variables related to happiness, such as economic status, education, and health.

### Subjective wealth and happiness

The association between income and happiness has been the most enduring debate in the literature on subjective wellbeing [43–45]. Studies showed positive and negative effects of wealth on happiness. Earlier studies reported various levels of correlation between income and happiness—in some cases, no relation at all [29, 44, 46]. Studies favoring the positive effects of wealth argued that higher income boosts purchasing power, expands affordable goods, and increases consumption, which leads to improved wellbeing [47]. Experimental studies showed that the increase in income significantly affects the level of happiness. For example, unconditional cash transfers from government or NGO increased recipients’ happiness in Zambia [48] and Kenya [49]. A study using a multilevel model with data from World Values Survey (*N* = 64,923, *k* = 81 nations) showed consistent results [50].

In support of the adverse effects of wealth on happiness, a review study noted that income only accounted for 4% of the variance of subjective wellbeing [51]. Higher-income is related to less daily sadness, but not to daily happiness [52]. More recent studies depicted time- and money-spending behaviors as variables that should be accounted for in the relationship between wealth and happiness [48, 53–57].

This study used subjective wealth as a proxy for objective wealth. Previous studies showed that subjective wealth is associated with objective wealth [58]. There is also a consensus among researchers that subjective wealth is a predictor of general happiness [58, 59].

### Education and happiness

The direct relationship between education and happiness is still unclear, though several studies found correlations between them [60–64]. Jongbloed [64] stated that higher education is significantly associated with happiness. One possible explanation is that higher education is associated with longer and healthier lives, successful marriages, higher quality of interpersonal relationships, and better opportunities on the labor market [62, 65]. Another study stated that non-monetary factors also play a role in the relationship between education and happiness, e.g., interpersonal networking and degree of cosmopolitanism. Better-educated people have broader social networks and involvement with the wider world, which is associated with happiness [66].

Nevertheless, an increasing number of studies established an insignificant or declined relationship between higher education and happiness [29, 62]. One study pointed out that income and occupation moderated the association between education and happiness [67]. When income and occupation were controlled, education had a negative effect on happiness [67]. A longitudinal study found similar results; participants with only secondary education (non-vocational) were healthier, happier, and wealthier when compared to other groups [68].

Education’s correlation with happiness seems to be affected by the country’s overall welfare, with high correlations in developing nations and low correlation in rich ones [69]. This is not because education breeds dissatisfaction but possibly due to scarcity of employment that matches the level of education or the fading of earlier advantages in the process of social equalizing [69].

### Health and happiness

The hypothesis that health affects happiness is widely accepted. One study found that self-rated health correlated significantly with subjective wellbeing [70]. A recent study based on Asian samples also concluded that self-rated health greatly affected happiness, especially when physical health problems occurred [71]. Another study, based on a community sample of older adults, concluded that health status is one of the most influential predictors of happiness [72]. However, disease severity has little effect on happiness. People with cancer can be happier than people with allergies. A higher relationship was found between happiness and the degree to which disease disrupts daily functioning [72].

**Fig. 1:**
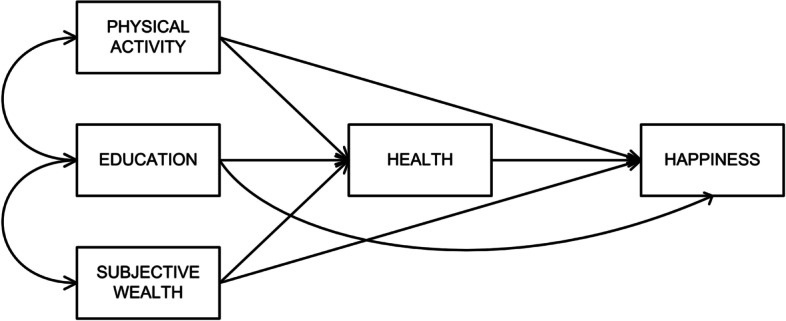
Hypothesized model of physical activity’s effects on happiness

Based on the context that physical activity’s psychological benefits are limited and mostly related to cognitive functions, there is a need to examine the effect of physical activity on other psychological functions, i.e., happiness. The present study proposed a model to investigate the association between physical activity and happiness. Based on the aforementioned studies, the model includes other related variables—subjective wealth, health, and education. The analysis also controlled for age and gender. Figure 1 shows the hypothesized relationship model between physical activity, subjective wealth, education, health, and happiness.

$${\displaystyle \begin{array}{c}{\mathcal{y}}_1=\upalpha +{\upgamma \mathcal{x}}_1+{\upgamma \mathcal{x}}_2+{\upgamma \mathcal{x}}_3+{\upzeta}_1\\ {}{\mathcal{y}}_2=\upalpha +{\upgamma \mathcal{x}}_1+{\upgamma \mathcal{x}}_2+{\upgamma \mathcal{x}}_3+{\mathcal{y}}_2\uppsi +{\upzeta}_2\end{array}}$$

Where 𝓎 is the endogenous variable (i.e., 𝓎_1_: health, 𝓎_2_: happiness), α is the intercept, γ is the regression coefficient, 𝓍 is the exogenous variable (i.e., 𝓍_1_: physical activity, 𝓍_2_: education, 𝓍_3_: subjective wealth), ψ is the residual variance of the endogenous variable, and ζ is the residual or error in the equation [73].

## Materials and methods

This study is a quantitative study using a structural equation modeling (SEM). SEM is a multivariate technique combining aspects of factor analysis and multiple regression that enables the researcher to simultaneously examine a series of interrelated dependence relationships among variables [74]. The data were from a longitudinal socioeconomic and health survey in Indonesia, the Indonesian Family Life Survey (IFLS). IFLS has been conducted five times, in 1993, 1998, 2000, 2007, and 2014–2015. IFLS sampling scheme stratified on provinces and urban/rural location and then randomly sampled within the strata. The sampling method considered the cultural and socioeconomic diversity in Indonesia and represent four most populated islands in Indonesia—Java, Sumatra, Kalimantan, and Sulawesi—containing 83% of the population. The first IFLS sampling frame were based on 1993 SUSENAS (National Socioeconomic Survey), which was based on 1990 census [75].

The IFLS surveys were reviewed and approved by IRBs in the United States and in Indonesia at Universitas Gadjah Mada (UGM). The ethical clearance number from RAND’s Human Subjects Protection Committee (RAND’s IRB) was s0064-06-01-CR01.

### Participants

From the total dataset, 12,051 participants were selected for the analysis. The gender composition was 47.4% male (*n* = 5711) and 52.6% female (*n* = 6340). Participant inclusion was based on: (1) ages 18 to 65, (2) completion the necessary variables in the questionnaire, and (3) not having a chronic disease (i.e., asthma, cancer or malignant tumor, memory-related disease, high-cholesterol).

### Variables

#### Physical activities

The IFLS (Indonesian Family Life Survey) questionnaire includes physical activity variables in Book IIIB Section KK (Health condition). There are nine questions in the section measuring activities type and their duration. Participants were asked to quantify their activity in the previous 7 days. The levels of physical activity were vigorous activities, moderate physical effort, and walking [76]. Vigorous activities were described as those that made participants breathe much harder than usual, such as heavy lifting, digging, plowing, aerobics, fast bicycling, and cycling with loads. Moderate physical activities were those that made participants breathe somewhat harder than usual, such as carrying light loads, bicycling at a regular pace, and mopping the floor. Walking included daily walking at work, at home, to travel from place to place, or in any other context related to recreation, sport, exercise, or leisure. The intensity of physical activities was measured by the duration per day (less than 30 minutes to more than 4 hours) and the number of days performed in the previous 7 days.

Physical activity data were converted to metabolic equivalent value (MET) according to the Compendium of Physical Activities [77]. One MET is equal to the energy spent when a person sits still. The MET for walking is from two to eight, depending on speed and obstacles. In this study, vigorous physical activities were categorized as multiple household tasks (vigorous) in the Compendium of Physical Activities, which equals four MET. Moderate physical activities were categorized as multiple household tasks (medium), which equals 3.5 MET, while walking was categorized as equal to 2.5 MET. The participants’ METs were afterward converted to MET minutes (MET × 60 seconds). The participants’ average MET minutes were 69.6 (*SD* = 55.5).

#### Subjective wealth

Question SW01 from Book IIIA of the IFLS questionnaire measured the subjective wealth of the participants, who were asked to rate their overall wealth from 1 ‘Poorest’ to 6 ‘Richest’. The average response was 2.86 (*SD* = .8).

#### Education

Participants were asked about their highest level of education (question DL6 Book IIIA). Among them, 6.6% had at least a bachelor’s degree, and 5.9% had a diploma degree (3 years of vocational education after high school). Table 1 shows the complete statistics of participants’ education levels.

**Table 1:** Descriptive statistics

Variables	*N* = 12,051
Age, mean (*SD*)	34.57 (11.3)
Gender, *n* (%)	
Male	5711 (47.4)
Female	6340 (52.6)
Education, *n* (%)	
≤ High school	10,777 (89.4)
Diploma (vocational degree)	405 (5.9)
Bachelor degree	772 (6.4)
Master degree	19 (0.2)
Subjective health, *n* (%)	
Unhealthy	13 (.1)
Somewhat unhealthy	1362 (11.3)
Somewhat healthy	9298 (77.2)
Very healthy	1378 (11.4)
Physical activity, mean (SD)	69.60 (55.5)
Happiness, mean (*SD*)	2.98 (.4)
Happiness, *n* (%)	
Very unhappy	29 (.2)
Unhappy	851 (7.1)
Happy	10,457 (86.8)
Very happy	714 (5.9)
Subjective wealth, mean (*SD*)	2.86(.8)
Subjective wealth, *n* (*%*)	
Perceived income ladder 1 & 2	3360 (27.9)
Perceived income ladder 3 & 4	8554 (71.0)
Perceived income ladder 5 & 6	137 (1.1)

#### Health

Participants’ responses to question KK01 in Book IIIB were used to indicate health. Participants were asked about the general condition of their health. More than 88.6% stated that they were somewhat healthy or very healthy. Table 1 presents the complete statistics of the participants’ responses.

#### Happiness

Participants’ responses to question SW12 of the IFLS Book IIIA were used to indicate happiness. Participants were asked to rate their happiness. The scale ranged from 1 ‘Very unhappy’ to 4 ‘Very happy.’ Participants’ average self-rated happiness was 2.98 (*SD* = .4). More than 91% of the participants were happy or very happy.

### Statistical analysis

In the present study, the model was tested using structural equation modeling or analysis of covariance structure. To assess the fitness of the model, it is necessary to report fit statistics such as *χ*^2^ value and degrees of freedom; the CFI (comparative fit index) or TLI (Tucker-Lewis Index); and RMSEA (root mean square error of approximation) [74, 78]. The threshold of fit indices was based on the recommendation of Hair et al. [74]. For a model with less than 12 observed variables and *n* more than 250, the suggested values are CFI ≥ .97 and RMSEA < .07 [74].

## Results

**Fig. 2:**
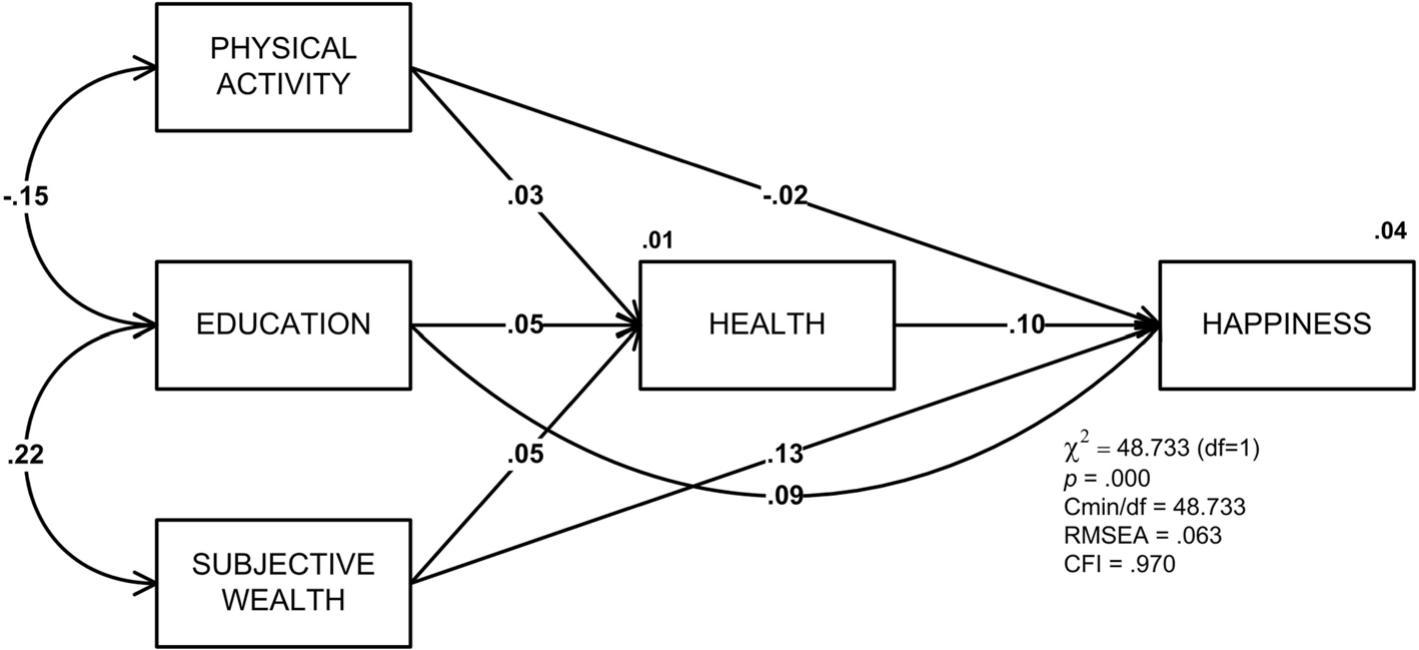
Final model of physical activity’s effects on happiness

When the hypothesized model was fitted to the data, the following fit indices resulted: *χ*^2^ (1, *N* = 12,051) = 48.733, *p* = .001, RMSEA = .063, CFI = .970. This fulfilled the requirements for a good model fit by Hair and colleagues [74]. Figure 2 depicts the structural model.

The analysis was controlled for participants’ gender. Studies show that gender difference is relevant in happiness studies. Table 2 shows the fit statistics of the final model by gender.

**Table 2:** Fit statistics of the model by gender and field of study

	N	χ^2^	*df*	RMSEA	CFI
Male	5711	14.557	1	.049	.985
Female	6340	28,250	1	.066	.964
Total data	12,051	48,733	1	.063	.970

As shown in Table 3, all standardized regression weights are positively significant except for the path from physical activity to happiness which is negatively significant. Table 3 and Fig. 2 show the mediating effects of health in the relationship between physical activity and happiness. The highest effect was found in the path from subjective wealth to happiness (*β* = .13), followed by health to happiness (*β* = .095), and education to happiness (*β* = .095). Education, subjective wealth, and physical activity positively affect health (*β* = .049, *β* = .048, and *β* = .025, respectively). The value above happiness (.04) in Fig. 2 is the square of multiple correlations and indicates the variability in happiness that is explained by other variables in the model. This means the variables in the model explained only 4% of the variability in happiness.

**Table 3:** Standardized regression weight of the predictors on happiness

	*β*	SE	CR	p*
Subjective wealth - > Health	.048	.006	5.133	.000
Physical activity - > Health	.025	.000	2.736	.006
Education - > Health	.048	.004	5.111	.000
Subjective wealth - > Happiness	.130	.004	14.263	.000
Health - > Happiness	.095	.007	10.678	.000
Education - > Happiness	.092	.003	9.911	.000
Physical activity - > Happiness	−.020	.000	−2.259	.024

*Note*. * one-tailed. *SE* Standard error, *CR* Critical ratio

## Discussion

Based on data from a large survey in Indonesia, the present study examined the relationship between physical activity, subjective wealth, health, education, and happiness. Based on the structural model, the overall analysis showed that the hypothesized model is a good fit for the dataset.

Further analysis found that physical activity did not directly affect happiness. Physical activity has a negative effect on physical activity. However, health conditions mediated the relationship between physical activity and happiness. This result is consistent with prior studies on the mediating effects of health on happiness [33, 79, 80]. Few studies have investigated the mediating effect of health in general populations [79]; most focused on older populations [80, 81]. The present study makes a noteworthy contribution since the dataset was based on a large sample from the general population of a developing country.

A recent review highlighted the mediating effects of health on the relationship between physical activity and happiness [33]. Engaging in physical activity contributes to the perception of good health, thus potentially improving happiness [79]. Other studies also showed an indirect association between physical activity and happiness, mediated by health and social functioning [80]. A possible explanation is that less physically active people, such as physically limited or ill older adults, face difficulty socializing with others. This leads to poor social functioning related to low levels of happiness [80].

The negative effect of physical activity on happiness may partly be explained by the participants’ diverse physical activity levels. A high level of physical activity is often associated with a blue-collar occupation [82], lower socioeconomic status, and a low level of happiness. Further analysis of the model by controlling the level of physical activity—including only participants who have recommended physical activity [83, 84]—supported this argument. After controlling the level of physical activity, the model showed a non-significant direct effect of physical activity on happiness.

Another contribution of the present study is the use of a structural model rarely used by previous studies investigating physical activity and happiness. Structural equation modeling is the appropriate method for evaluating a series of simultaneous hypotheses about the impacts of latent and manifest variables on other variables while taking measurement errors into account [74, 85]. Therefore, the present study’s structural modeling gives a better understanding of the relationships between physical activity, education, subjective wealth, health, and happiness.

In the present study, the highest regression coefficient was in the path from subjective wealth to happiness. This finding supports prior studies regarding subjective wealth’s positive effects on happiness [43, 45, 50]. Classic economic theories posit that higher-income boosts purchasing power, expands affordable goods, and increases consumption, leading to improved wellbeing [47]. One study showed that wealth is positively and significantly associated with happiness in low-income and high-income countries; however, higher effects were found in the former than in the latter [50]. This relates to the ceiling effect on the relationship between income and happiness—i.e., income satiation. After reaching a certain level, income no longer affects happiness [57].

The structural model also showed a significant positive effect of education on happiness. These results support previous research on the association between education and happiness [60, 61, 63–65, 86]. However, an increasing number of studies depict an insignificant relationship between education and happiness [67–69]. One possible explanation for this is that most of these studies use life satisfaction as a proxy for happiness [86], which is inaccurate because it only measures the evaluative dimension of happiness, excluding the hedonic and eudaimonic dimensions [86].

Several limitations must be noted regarding the present study. First, most variables were based on subjective self-report, which is open to biases. Responses might be affected by social norms [29]; e.g., participants might under-report their wealth because humbleness is valued under Indonesian social norms. The second is that, evengthough SEM previously used to indicate a causality evidence [87], the cross-sectional design of this study prohibiting evidence for directionality relations. Future studies should consider using objective measurements—i.e., for health and wealth. The use of latent variables should also be considered. Including variables such as religiosity and social relationships would be interesting, especially in the Indonesian context.

## Conclusion

This study suggests that health mediates physical activity’s effects on happiness. Active individuals would have better health compared to their sedentary counterparts. Health condition, as suggested by the model, was one of the biggest predictors of happiness. The fact that physical activity positively affects happiness should also motivate Indonesians to engage in more active lifestyles. This is important since the national health survey revealed that more than one-third (33.5%) of Indonesians did not get enough physical activity [88].

## Data Availability

This study used IFLS Public Use Data which are available in RAND website: https://www.rand.org/well-being/social-and-behavioral-policy/data/FLS/IFLS/access.html.
